# Low expression of c-Myc protein predicts poor outcomes in patients with hepatocellular carcinoma after resection

**DOI:** 10.1186/s12885-018-4379-5

**Published:** 2018-04-24

**Authors:** Fei Ji, Zhi-heng Zhang, Yi Zhang, Shun-Li Shen, Qing-Hua Cao, Long-Juan Zhang, Shao-Qiang Li, Bao-Gang Peng, Li-Jian Liang, Yun-Peng Hua

**Affiliations:** 1 Pediatric Surgery, The First Affiliated Hospital, Sun Yat-sen University, Guangzhou, 510080 People’s Republic of China; 2 Organ Transplant Center, The First Affiliated Hospital, Sun Yat-sen University, Guangzhou, 510080 People’s Republic of China; 3 Department of Hepatobiliary Surgery, The Third Affiliated Hospital, Sun Yat-sen University, Guangzhou, 510630 People’s Republic of China; 4 Department of Liver Surgery, The First Affiliated Hospital, Sun Yat-sen University, Guangzhou, 510080 People’s Republic of China; 5 Department of Pathology, The First Affiliated Hospital, Sun Yat-sen University, Guangzhou, 510080 People’s Republic of China; 6 Laboratory of Surgery, The First Affiliated Hospital, Sun Yat-sen University, Guangzhou, 510080 People’s Republic of China

**Keywords:** C-MYC, Oncogene, Hepatocellular carcinoma, Prognosis, Biomarkers

## Abstract

**Background:**

Embryonic Liver Fodrin (ELF) is an adaptor protein of transforming growth factor (TGF-β) signaling cascade. Disruption of ELF results in mislocalization of Smad3 and Smad4, leading to compromised TGF-β signaling. c-Myc is an important oncogenic transcription factor, and the disruption of TGF-β signaling promotes c-Myc-induced hepatocellular carcinoma (HCC) carcinogenesis. However, the prognostic significance of c-Myc in HCC is less understood

**Methods:**

The expression of c-Myc protein and mRNA were measured by immunohistochemistry (IHC) and qRT- PCR, respectively. IHC was performed to detect TGF-β1 and ELF expression in HCC tissues. Their relationship with clinicopathological factors and overall survival (OS) and disease free survival (DFS) were examined.

**Results:**

The expression of c-Myc protein and mRNA in HCC tissues were significantly higher in HCC area than those in normal liver tissues. However, the expression were low compared with those adjacent to HCC area. c-Myc protein was independently predictive of DFS and OS, and it was negatively correlated with tumor size (*P* = 0.031), tumor number (*P* = 0.038), and recurrence (*P* = 0.001). Low c-Myc expression was associated with short-term recurrence and poor prognosis. The predictive value of c-Myc combined with TGF-β1 or/and ELF was higher than that of any other single marker. Low c-Myc, high TGF-β1 or/and low ELF expression was associated with the worst DFS and OS.

**Conclusions:**

Low expression of c-Myc protein predicts poor outcomes in patients with HCC with hepatectomy. The combination of the expression of c-Myc, TGF-β1, and ELF can be used to accurately predict outcomes of patients with HCC.

## Background

Hepatocellular carcinoma (HCC) is a very common and serious malignancy with high morbidity and high mortality. It is the sixth most common cancer and the third common cause of cancer-related death worldwide [1–3]^.^ Though most cases of HCC happen in Eastern Asia and sub-Saharan Africa, the incidence in some developed countries such as Japan, UK, France, and USA is increasing [4]^.^ The increase of incidence and the lack of effective treatment for HCC have made the disease a major health problem [5]. Therefore, it is important to understand the mechanisms of HCC to find the effective biomarkers and to develop therapies.

c-Myc, an important oncogenic transcription factor, locates on chromosome 8q24.1, and involves in cell proliferation, apoptosis, differentiation and metabolism because it participates in the regulation of 15% of all genes in the human genome, including microRNAs [6–12]. Furthermore, as a powerful cancer-promoting oncogene, c-Myc has been implicated in the pathogenesis of kinds of malignant tumors, including human solid tumor, leukemia and lymphoma, along with animal tumors [13–17]. Similarly, c-Myc is also identified as a reasonable gene driver and a central regulator of malignant transformation in primary stages of HCC carcinogenesis using gene expression profiling of cirrhotic and dysplastic nodules, and early HCC [18]. In addition, c-Myc gene is commonly found to be overexpressed in advanced HCC tissues and derived HCC cell lines too [19, 20]. Moreover, regression of invasive HCC in animal models could be induced by the inactivation of c-Myc gene, followed by up-regulation of liver cell and liver stem cell markers, and down-regulation of tumor markers [21].

Paradoxically, c-Myc is also found to induce apoptosis and cell senescence, and represent tumor-suppressive through activation of tumor suppressor p53 directly or indirectly [12, 17]. The recent studies indicate that tumorigenesis in mouse models of c-Myc oncogenesis was accelerated by inactivation of the ARF-MDM2-p53 pathway. c-Myc can also change the balance of pro- and anti-apoptotic factors to prime the cells for apoptosis and death [6, 22, 23]. Pelengaris et al. [24] used a switchable form of the c-Myc protein to make a reversible transgenic model of pancreatic β cell oncogenesis, and found that loss of c-Myc protein up-regulated Bcl-xL expression and suppressed apoptosis, finally resulted in a full spectrum of tumor development, even distant metastasis. Murakami et al. [25] and Lee et al. [26] found that c-Myc-induced HCC was marked by a relatively higher degree of cell differentiation, less aggressiveness, a lower rate of genomic instability, and a longer time for carcinogenesis. Other studies have also reported that the level of c-Myc expression determined its oncogenic potential, indicated that low c-Myc expression induced cell proliferation and oncogenesis, whereas high c-Myc expression activated pro-apoptotic programs and accelerated apoptosis [27–29].

In this study, we investigated the prognostic value of c-Myc expression in in patients with HCC undergoing curative resection. In addition, it is well-known that overexpression of TGF-β1 correlates with carcinogenesis and progression of HCC. We have observed that the high level of TGF-β1 and low level of ELF (a Smad3/4 adaptor protein) predicted the poor outcome in patients with HCC [30–32]. Santoni-Rugiu et al. [33] indicated that disruption of TGF-β1 signaling promoted c-Myc- induced HCC carcinogenesis by TGF-α as well. Therefore, this study further verified the more accurate predictive parameters of HCC using the combination of c-Myc with TGF-β1, and ELF.

## Methods

### Study population

This study was approved by the Ethics Review Committee of the First Affiliated Hospital of Sun Yat-sen University. Eighty-four patients with HCC receiving hepatectomy at our center from June 2007 to October 2009 were included in this study. The inclusion criteria were patients: 1) with tumor-node-metastasis (TNM) stage I, II, IIIA or IIIB (International Union Against Cancer, the seventh edition); 2) with Child-Pugh class A and class B hepatic function; 3) aged 18–80; and 4) provided written informed consent. The exclusion criteria included patients: 1) with TNM stage IIIC, IVA, or IVB; 2) with Child-Pugh class C hepatic function; 3) with second malignancy or history of second malignancy within 5 years; 4) with perioperative dysfunction of vital organs; 5) with percutaneous ablation, 6) transcatheter arterial chemoembolization (TACE); and 7) received chemotherapy, or radiotherapy within 1 month post-operation. All patients were postoperatively followed-up as described in our previous study [30]. Normal liver tissues, HCC adjacent tissues, and HCC tissues were collected and prepared as described in previous study [30].

### Immunohistochemical analysis

Immunohistochemical analysis was performed as previously described [30]. Rabbit polyclonal anti-c-Myc antibody (ab32072, Abcam, USA) was used in the immunohistochemical analysis (1:100 dilution).

### Evaluation of Immunohistochemical staining

The immunohistochemical staining in the tissue was scored independently by 2 pathologists blinded to the patients and clinical data, by applying a semiquantitative immunoreactivity score (IRS) reported elsewhere [30]. Category A documented the intensity of immunostaining as 0–3 (0, negative; 1, weak; 2, moderate; 3, strong). Category B documented the percentage of immunoreactive cells as 0 (less than 5%), 1 (6%–25%), 2 (26%–50%), 3 (51%–75%), and 4 (76%–100%). Multiplication of category A and B resulted in an IRS ranging from 0 to 12 for each tumor or non-tumor. Sections with a total score of 0 or 1 or 2 were defined as negative (−), score of 3 or 4 were defined as weakly positive (+), score of 6 or 8 were defined as moderately positive (++), score of 9 or 12 were defined as strongly positive (+++). For categorical analyses, the immunoreactivity was graded as low level (total score < =4) or high level (total score > 4).

### Real -time quantitative polymerase chain reaction (qRT-PCR)

Fresh HCC tissues (*n* = 29), HCC adjacent tissues (n = 29), and normal liver tissues (*n* = 4) were collected immediately after resection for detecting c-Myc mRNA expression. RNA was extracted by TRIzol reagent (Thermo Fisher Scientific, MA, USA) according to manufacturer’s protocol. The cDNA was synthesized by cDNA synthesis kit (Takara Biotechnology, Japan) in accordance with manufacturer’s instructions. The cDNA was used as templates for qRT-PCR by the SYBR Green PCR kit (Toyobo, Japan) in the Bio-rad IQ5 PCR system (Bio-Rad, CA). GAPDH was used as an internal control. The relative gene expressions were quantified and analyzed. The primers were obtained from Genecopia Company (Guangzhou, China).

### Statistical analysis

Normally distributed Continuous variables were compared by Student’s t-test. Categorical variables were compared with Chi-square test. Disease free survival (DFS) was defined as the interval from the surgery to recurrence, while overall survival (OS) was defined as the interval from surgery to the HCC-associated death. The univariate, multivariate and Kaplan–Meier method was used to analyze the survival rates [34], and the equivalences of the survival curves were compared among groups by log-rank test. A *P*-value < 0.05 would be regarded as statistical significance in each test. All analyses were performed using SPSS v 13 software (Chicago, IL, USA).

## Results

### The expression of c-Myc protein and mRNA in HCC and adjacent tissues

**Fig. 1:**
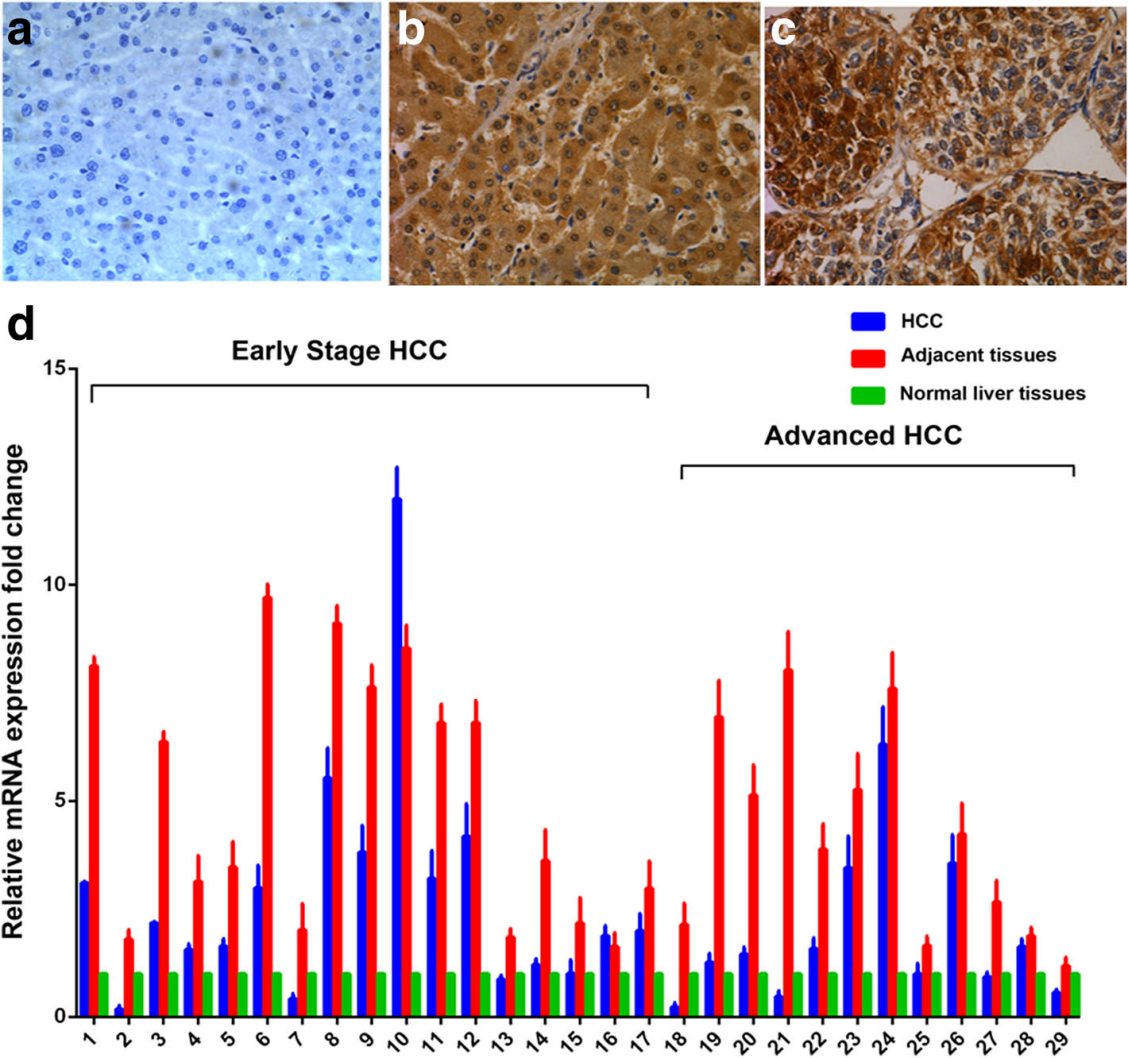
Expression of c-Myc. Immunohistochemical staining in different tissues is shown. Normal liver tissues (**a**), HCC adjacent tissues (**b**), HCC tissues (**c**) (original magnification × 400), and expression of c-Myc mRNA (**d**)

The expression of c-Myc in normal liver tissues was negative (0/20). However, c-Myc protein was expressed in 78.5% (66/84) in HCC tissues and in 82.1% (69/84) in adjacent tissues, and these rates were significantly different from that of normal liver tissues (*P* <  0.001). In adjacent tissues and HCC tissues, the high expression (++ to +++) rates for c-Myc were 65.5% (55/84) and 59.5% (50/84), respectively, and there were no statistical difference between them (*P* = 0.426) (Table 1, Fig. 1).

**Table 1:** The expression of c-Myc in HCC

Group	N	Expression of C-MYC	
		High (++~+++)	Low (−~+)
Normal liver tissues	20	0(0.0%)	20(100.0%)
Adjacent tissues^a^	84	55(65.5%)	29 (34.5%)
HCC tissues^a^	84	50(59.5%)	34(40.5%)

^a^ compared with Normal liver tissues, *P* < 0.001 (by chi-square test)

Similarly, we observed that the levels of c-myc mRNA in HCC tissues and in adjacent tissues were higher than that in normal liver tissues (*P* = 0.002 and P <  0.001, respectively). Interestingly, we found the level of c-myc mRNA in HCC tissues was remarkably lower than those in the corresponding adjacent tissue (P <  0.001). HCC tissues were divided into two groups in terms of TNM stage: Early stage HCC (TNM stage I and tumor size less than 10 cm, *n* = 17), and advanced HCC (TNM stage II and III, *n* = 12). There was no striking difference in c-myc mRNA expression between the early stage HCC and advanced HCC (Fig. 1).

### Correlation between 15 clinicopathological characteristics and c-Myc protein expression in HCC and adjacent tissues

To better understand the importance of the prognostic value of c-Myc protein expression, we first analyzed the relationships between 15 clinicopathological characteristics and c-Myc protein expression in cancer tissues (Table 2). The results showed that the expression of c-Myc protein in tumor tissues was not associated with age, sex, serum HBsAg positivity, serum alanine aminotransferase (ALT), and AFP level. There was also no relation between c-Myc expression and tumor differentiation, TNM stage, completeness of tumor encapsulation, and postoperative complications. However, the level of c-Myc protein in HCC tissues was positively correlated with cirrhosis (*P* = 0.042), but negatively correlated with platelet (PLT) count (*P* = 0.036), tumor number (*P* = 0.038), portal vein tumor thrombosis (PVTT) (*P* = 0.019), tumor size (*P* = 0.031), and recurrence (*P* = 0.002).

**Table 2:** Correlation between c-Myc and the clinicopathological characteristics in HCC tissues and adjacent tissues

Variables	Cases	c-Myc expression in HCC tissues		*P* value	c-Myc expression in adjacent tissues		*P* value
		Low	High		Low	High	
Age(yrs)							
> =60	16	4(25.0%)	12(75.0%)	0.161	3(18.8%)	13(81.2%)	0.140
< 60	68	30(44.1%)	38(55.9%)		26(38.2%)	42(61.8%)	
Sex							
Male	68	29(42.6%)	39(57.4%)	0.403	21(30.9%)	47(69.1%)	0.148
Female	16	5(31.3%)	11(68.7%)		8(50.0%)	8(50.0%)	
HbsAg							
Positive	72	30(41.7%)	42(58.3%)	0.586	25(34.7%)	47(65.3%)	0.925
Negative	12	4(33.3%)	8(66.7%)		4(33.3%)	8(66.7%)	
ALT(U/L)							
≥ 80	9	3(33.3%)	6(66.7%)	0.644	5(55.6%)	4(44.4%)	0.160
<80	75	31(41.3%)	44(58.7%)		24(32.0%)	51(68.0%)	
PLT(×10^9^)							
> 100	74	33(44.6%)	41(55.4%)	0.036	24(32.4%)	50(67.6%)	0.273
≤ 100	10	1(10.0%)	9(90.0%)		5(50.0%)	5(50.0%)	
Cirrhosis							
Yes	64	22(34.4%)	42(65.6%)	0.042	19(29.7%)	45(70.3%)	0.095
No	20	12(60.0%)	8(40.0%)		10(50.0%)	10(50.0%)	
AFP(ug/L)							
≥ 20	48	22(45.8%)	26(51.2%)	0.248	19(39.6%)	29(60.4%)	0.260
<20	36	12(33.3%)	24(66.7%)		10(27.8%)	26(72.2%)	
Tumor size (cm)							
≥ 5	50	25(50.0%)	25(50.0%)	0.031	19(38.0%)	31(62.0%)	0.416
<5	34	9(26.5%)	25(73.5%)		10(29.4%)	24(70.6%)	
Tumor number							
Single	62	21(33.9%)	41(66.1%)	0.038	20(32.3%)	42(67.7%)	0.463
Multiple	22	13(59.1%)	9(40.9%)		9(40.9%)	13(59.1%)	
Differentiation							
I-II	62	24(38.7%)	38(61.3%)	0.580	25(40.3%)	37(59.7%)	0.061
III	22	10(45.5%)	12(54.5%)		4(18.2%)	18(81.8%)	
TNM stage							
I	55	20(36.4%)	35(63.6%)	0.290	18(32.7%)	37(67.3%)	0.633
II-III	29	14(48.3%)	15(51.7%)		11(37.9%)	18(62.1%)	
PVTT							
Yes	11	8(72.7%)	3(27.3%)	0.019	4(36.4%)	7(63.6%)	0.890
No	73	26(35.6%)	47(64.4%)		25(34.2%)	48(65.8%)	
Tumor Encapsulation							
Complete	64	23(35.9%)	41(64.1%)	0.130	17(26.6%)	47(73.4%)	0.006
None	20	11(55.0%)	9(45.0%)		12(60.0%)	8(40.0%)	
Recurrence							
Yes	58	30(51.7%)	28(48.3%)	0.002	21(36.2%)	37(63.8%)	0.628
No	26	4(15.4%)	22(84.6%)		8(30.8%)	18(69.2%)	
Complication							
No	73	27(37.0%)	46(63.0%)	0.177	27(37.0%)	46(63.0%)	0.221
Yes	11	7(63.6%)	4(36.4%)		2(18.2%)	9(81.8%)	

*AFP* Alpha-fetoprotein, *ALT* alanine aminotransferase, *HBsAg* hepatitis B surface antigen, *PLT* platelet, *PVTT* portal vein tumor thrombi

In tissues adjacent to HCC, high expression of c-Myc was only associated with complete tumor encapsulation (*P* = 0.006).

### Independent prognostic factors of HCC

In order to discern the factors related to DFS and OS, c-Myc and clinicopathological factors were assessed by univariate analysis and the Multivariate analysis. The univariate analysis indicated that significant prognostic factors for DFS were PVTT, tumor number, tumor encapsulation, blood transfusion, blood loss, operative time, TNM stage, and c-Myc expression. The significant prognostic factors for OS were PVTT, tumor number, resection margin, tumor size, tumor differentiation, TNM stage, blood transfusion, blood loss, operative time and c-Myc expression (all *P* <  0.05) (Table 3). Multivariate analysis indicated that blood transfusion, TNM stage and c-Myc expression were significantly independent prognostic factors for both DFS and OS (all P <  0.05). (Table 4).

**Table 3:** Prognostic factors for DFS and OS by univariate analysis

Variables	*n*	DFS			*P*	OS			P
		1-yr	3-yrs	5-yrs		1-yr	3-yrs	5-yrs	
Sex									
Male	68	47.1%	36.8%	30.5%	0.433	79.4%	50.0%	41.2%	0.478
Female	16	56.3%	37.5%	37.5%		87.5%	56.3%	50.0%	
Age(yrs)									
< 60	68	45.6%	32.4%	27.7%	0.104	82.4%	48.5%	39.7%	0.391
≥ 60	16	62.5%	56.3%	49.2%		75.0%	62.5%	56.3%	
PLT(×10^9^)									
<100	10	80.0%	60.0%	60.0%	0.053	100%	80.0%	70.0%	0.076
≥ 100	74	44.6%	33.8%	28.0%		78.4%	47.3%	39.2%	
HbsAg									
Positive	72	47.2%	38.9%	33.0%	0.700	79.2%	50.0%	44.4%	0.882
Negative	12	58.3%	25.0%	25.0%		91.7%	58.3%	33.3%	
AFP(μg/L)									
<20	36	52.8%	38.9%	38.9%	0.336	83.3%	50.0%	44.4%	0.750
≥ 20	48	45.8%	35.4%	26.3%		79.2%	52.1%	41.7%	
Ascites									
No	68	52.9%	39.7%	35.0%	0.093	83.8%	51.5%	44.1%	0.551
Yes	16	31.3%	25.0%	18.8%		68.8%	50.0%	37.5%	
Cirrhosis									
No	24	45.0%	35.0%	30.0%	0.685	95.0%	60.0%	45.0%	0.485
Yes	60	50.0%	37.5%	32.4%		76.6%	48.4%	42.2%	
Tumor number									
Single	62	59.7%	43.5%	36.7%	0.002	85.5%	61.3%	51.6%	< 0.001
Multiple	22	18.2%	18.2%	18.2%		68.2%	22.7%	18.2%	
PVTT									
No	73	54.8%	41.1%	35.3%	< 0.001	87.7%	56.2%	47.9%	< 0.001
Yes	11	9.1%	9.1%	9.1%		36.4%	18.2%	9.1%	
Tumor size (cm)									
< 5	34	64.7%	47.1%	41.2%	0.055	97.1%	67.6%	52.9%	0.040
≥ 5	50	38.0%	30.0%	25.7%		70.0%	40.0%	36.0%	
Tumor Encapsulation									
None	20	30.0%	25.0%	15.0%	0.013	60.0%	45.0%	30.0%	0.081
Complete	64	54.7%	40.6%	37.3%		87.5%	53.1%	46.9%	
Resection margin									
<2 cm	45	40.0%	26.7%	24.4%	0.106	80.0%	40.0%	28.9%	0.007
≥ 2 cm	39	59.0%	48.7%	40.5%		82.1%	64.1%	59.0%	
Complication									
No	73	49.3%	38.4%	32.8%	0.407	84.9%	53.4%	45.2%	0.095
Yes	11	45.5%	27.3%	27.3%		54.5%	36.4%	27.3%	
Tumor differetiation									
I-II	62	54.8%	41.9%	35.0%	0.212	85.5%	56.5%	48.4%	0.037
III-IV	22	31.8%	22.7%	22.7%		68.2%	36.4%	27.3%	
Blood inflow occlusion									
Yes	31	41.9%	29.0%	20.7%	0.182	77.4%	48.4%	35.5%	0.300
No	53	52.8%	41.5%	37.7%		83.0%	52.8%	47.2%	
Blood transfusion									
Yes	25	20.0%	4.0%	4.0%	< 0.001	56.0%	12.0%	4.0%	< 0.001
No	59	61.0%	50.8%	43.7%		91.5%	67.8%	59.3%	
Blood loss (mL)									
≤ 1000	73	54.8%	42.5%	36.7%	< 0.001	86.3%	58.9%	49.3%	< 0.001
> 1000	11	9.1%	0.0%	0.0%		45.5%	0.0%	0.0%	
Operative time(min)									
≤ 180	42	66.7%	54.8%	47.3%	< 0.001	92.9%	73.8%	64.3%	< 0.001
> 180	42	31.0%	19.0%	16.7%		69.0%	28.6%	21.4%	
TNM stage									
I	55	60.0%	43.6%	39.7%	0.002	90.9%	60.0%	52.7%	0.001
II-III	29	27.6%	24.1%	17.2%		62.1%	34.5%	24.1%	
c-Myc									
Low	34	17.6%	11.8%	11.8%	< 0.001	64.7%	20.6%	14.7%	< 0.001
High	50	70.0%	54.0%	45.9%		92.0%	72.0%	62.0%	

*AFP*Alpha-fetoprotein, *ALT* alanine aminotransferase, *HBsAg* hepatitis B surface antigen, *PLT* platelet, *PVTT* portal vein tumor thrombi

**Table 4:** Prognostic factors for disease-free and overall survival by the multivariate Cox proportional hazards regression model

*Variables*	*DFS*			*OS*		
	HR	95%CI	*P*	HR	95%CI	*P*
Blood transfusion	1.922	1.017–3.635	0.044	2.856	1.465–5.567	0.002
TNM stage	2.143	1.220–3.766	0.008	2.191	1.169–4.108	0.014
c-Myc	0.364	0.194–0.686	0.002	0.284	0.148–0.548	< 0.001

*HR* hazard ratio, CI confidence interval

### Low expression of c-Myc predicts short-term recurrence and the poor outcomes in patients with HCC.

**Fig. 2:**
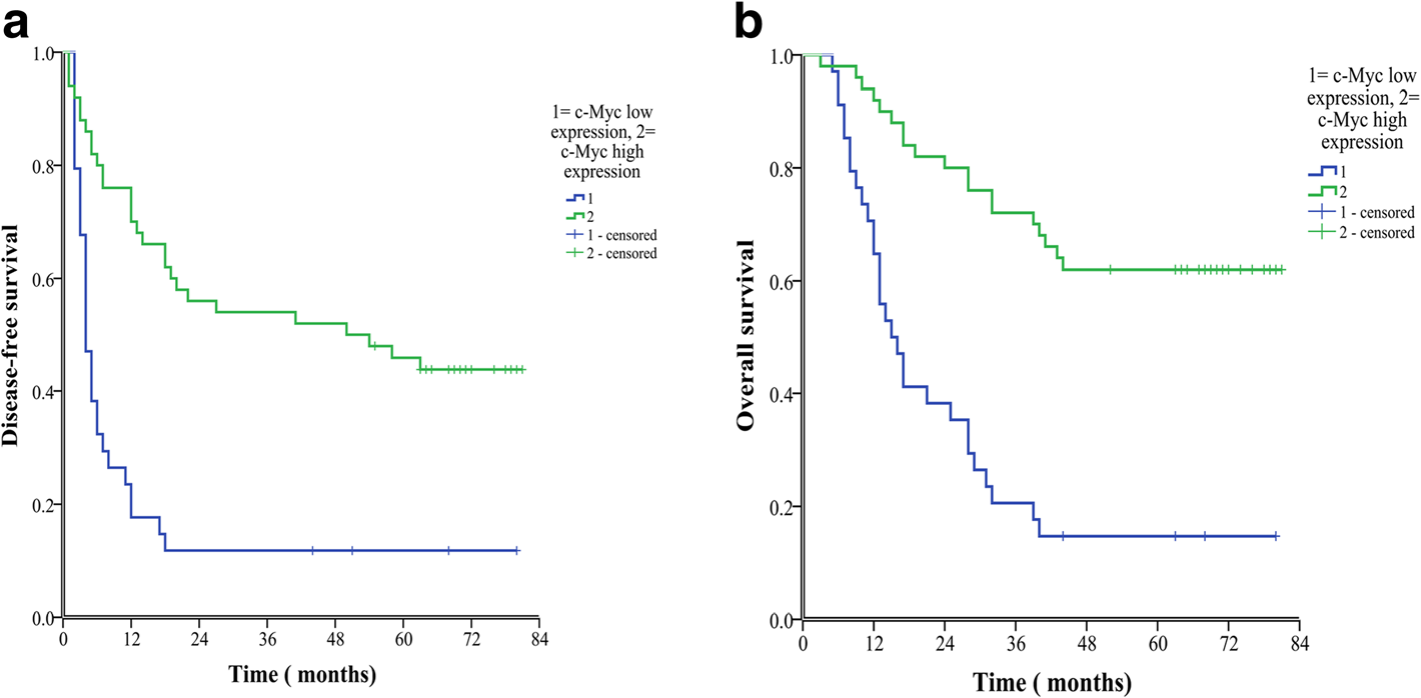
Kaplan-Meier curves are shown for time to disease recurrence (**a**, *P* < 0.001, log-rank test) and overall survival (**b**, *P* < 0.001, log-rank test) among patients with high or low intratumoral c-Myc expression

All patients were divided into 2 groups in terms of their c-Myc expression profiles: high-expression (*n* = 50) and low-expression (*n* = 34). Analysis showed that c-Myc expression level was positively correlated with DFS and OS. The 1-, 3-, and 5-year DFS rates in the high c-Myc expression group were significantly higher than those of low expression group (70.0%, 54.9%, 45.9% vs. 17.6%, 11.8%, 11.8%, respectively, *P* <  0.001) (Fig. 2a). In addition, the 1-, 3-, and 5-year OS rates of the high expression group were still markedly higher than those of low expression group (92.0%, 72.0%, 62.0% vs. 64.7%, 20.6%, 14.7%, respectively, P <  0.001) (Fig. 2b).

**Fig. 3:**
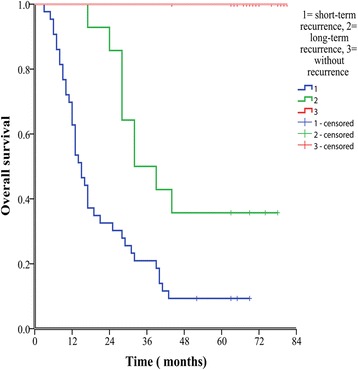
Kaplan-Meier curves are shown for time to overall survival among patients with recurrence in different times (*P* < 0.001, log-rank test)

In the current study, the total recurrence rate was 67.9% (57/84); however, 43 cases of recurrence happened in the first postoperative year. The 1-, 3-, and 5-year OS rates of the in patients with HCC with a recurrence in first year (62.8%, 20.9%, 9.3%, respectively) were obviously lower than that of patients with a recurrence after 1 years (100%, 50.0%, 35.7%, respectively, P <  0.001), or those without recurrence (100%,100%, 100%, P <  0.001). There was also a marked difference rates between patients with a recurrence after 1 year and those without a recurrence *(*P <  0.001, Fig. 3).

We then analyzed the association between c-Myc expression and recurrence time, and found that the proportion of patients with low c-Myc expression with short term recurrence (62.8%) was higher than that in patients with a long term recurrence (14.3%, *P* = 0.002) or without recurrence (14.8%, *P* < 0.001). However, there was no apparent difference between patients with a long term recurrence and patients with no recurrence (*P* = 0.964) (Table 5).

**Table 5:** The correlationship between c-Myc and recurrence

Recurrence	N	c-Myc expression	
		High	Low
0–1(yrs) ^a,b^	43	16(37.2%)	27 (62.8%)
> 1(yrs)^c^	14	12(85.7%)	2 (14.3%)
No recurrence	27	23(85.2%)	4(14.8%)

^a^ compared with > 1 year recurrence, *P* = 0.002

^b^ compared with no recurrence, *P* < 0.001.

^c^compared with no recurrence, *P* = 0.964 (by chi-square test)

### Combining c-Myc expression with TGF-β1 or/and ELF reveals improved prognostic accuracy for HCC

**Fig. 4:**
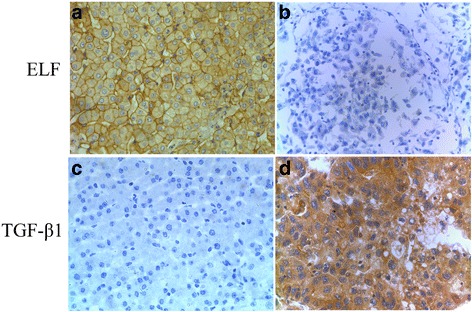
Expression of ELF and TGF-β1 immunohistochemical staining in different tissues is shown. Normal liver tissues (**a**,**c**), HCC tissues (**b**,**d**) (original magnification × 400)

As described in our previous study [30], high level of ELF protein (a Smad3/4 adaptor protein) was observed in normal liver tissues and low level of ELF protein in HCC tissues (Fig. 4a, b). In contrast, high level of TGF-β1 protein was found in human HCC tissues and low level of TGF-β1 protein in normal liver tissues (Fig. 4c, d). Then, we demonstrated a significant negative correlation between c-Myc and TGF-β1 (*r* = − 0.228, *P* = 0.037, Table 6), and a significant positive correlation between c-Myc and ELF (*r* = 0.217, *P* = 0.048, Table 7). We also analyzed the prognostic value of combined TGF-β1 and c-Myc levels for HCC. We divided patients into the following four groups: TGF-β1 high expression/c-Myc high expression (ThMh), TGF-β1 low expression/c-Myc high expression (TlMh), TGF-β1 high expression/c-Myc low expression (ThMl), TGF-β1 low expression /c-Myc low expression (TlMl). Analysis exhibited that the TlMh group had the best OS. The DFS and the TlMl groups had the second best rates, followed by the ThMh group, and the worst prognosis was seen in the ThMl group.

**Table 6:** The correlationship between c-Myc and TGF-β1 in HCC

*c-Myc*	*TGF-β1*			*r*	***P value***
	+++	++	-~+		
+++	7	5	12	−0.228	0.037
++	9	3	15		
-~+	15	11	7

**Table 7:** The correlationship between c-Myc and ELF in HCC

*c-Myc*	*ELF*			*r*	*P value*
	+++	++	-~+		
+++	12	1	11	0.217	0.048
++	4	11	12		
-~+	7	5	21		

**Fig. 5:**
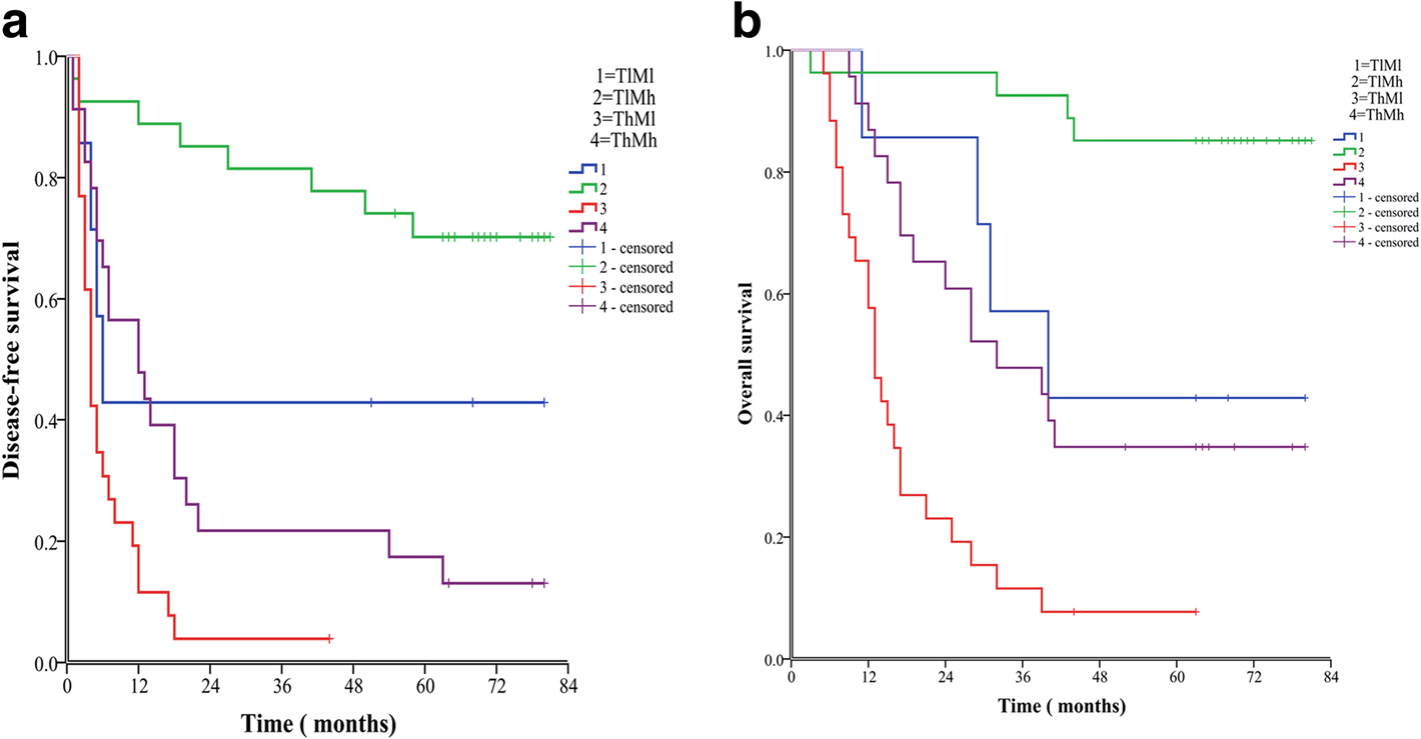
The combination of c-Myc and TGF-β1 was found to enhance prognostic accuracy for HCC. Disease-free survival curves (**a**, *P* < 0.001, log-rank test) and overall survival curves (**b**, *P* < 0.001, log-rank test)

The 1-, 3-, and 5-year DFS rates of the TlMh group (88.9%, 81.5%, 70.2%, respectively) were markedly higher than those of ThMh group (47.8%, 21.7%, 17.4%, respectively, *P* < 0.001) and the ThMl group (11.5%, 3.8%, 3.8%,respectively, P < 0.001). The 1-, 3-, and 5-year OS rates of the TlMh group (96.3%, 92.6%, 85.2%,respectively) were obviously higher than those of the ThMh group (87.0%, 47.8%, 34.8%,respectively, P < 0.001) and the ThMl group (57.7%, 11.5%, 7.7%,respectively, P < 0.001) (Fig. 5a, b). Furthermore, the 1-, 3- and 5-year DFS rates of the ThMl group were remarkably lower than those of the ThMh group (*P* = 0.004) and TlMl group (42.9%, 42.9%, 42.9%, respectively, P = 0.004). The 1-, 3-, and 5-year OS rates of the ThMl group were markedly lower than those of the ThMh group (*P* = 0.001) and TlMl group (42.9%, 42.9%, 2.9%, respectively, *P* = 0.012). Moreover, there was a significant difference of OS between the TlMh group and TlMl group (*P* = 0.007) (Fig. 5a, b). Overall, the results showed that the combination of TGF-β1 elevation and c-Myc reduction in HCC tissues seems to be predictive of poorest prognosis.

Second, we divided patients into another four groups in order to analyze the prognostic value of combined ELF and c-Myc levels for patients with HCC: ELF high expression/c-Myc high expression (EhMh), ELF low expression/c-Myc high expression (ElMh), ELF high expression/c-Myc low expression (EhMl), ELF low expression/c-Myc low expression (ElMl). The analysis indicated that the EhMh group had the best OS and DFS rates, the ElMh group the second best, followed by the EhMl group, and the ElMl group had the worst prognosis.

**Fig. 6:**
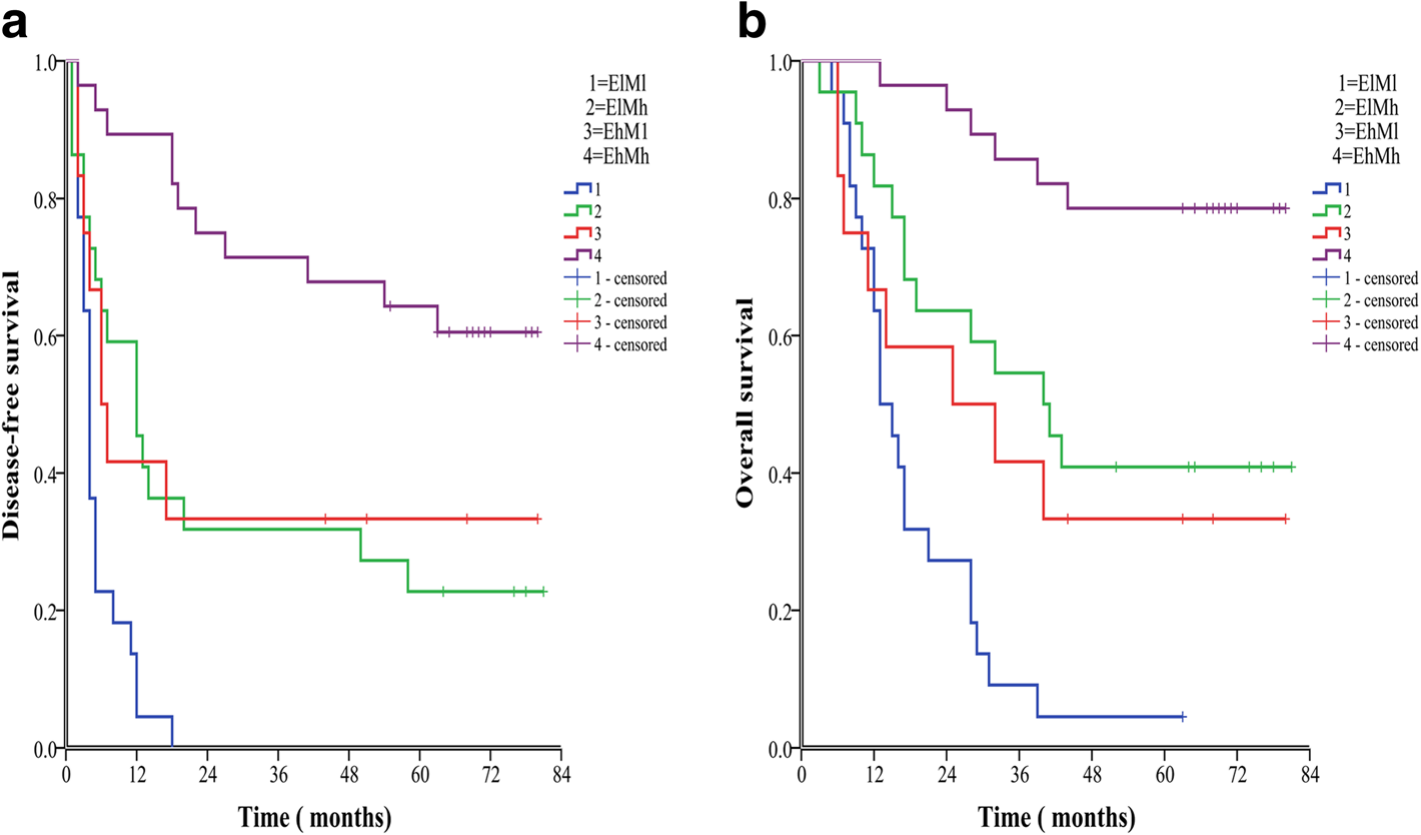
The combination of c-Myc and ELF was found to enhance prognostic accuracy for HCC. Disease-free survival curves (**a**, *P* < 0.001, log-rank test) and overall survival curves (**b**, *P* < 0.001, log-rank test)

The 1-, 3-, and 5-year DFS rates of EhMh group (89.3%, 71.4%, 64.3%, respectively) were markedly higher than those of the ElMh group (45.5%, 31.8%, 22.7%, respectively, *P* < 0.001) and the ElMl group (4.5%, 0%, 0%, respectively, P < 0.001). The 1-, 3-, and 5-year OS rates of the EhMh group (100%,85.7%, 78.6%, respectively) were significantly higher than those of the EhMl group (66.7%, 41.7%, 33.3%,respectively, P < 0.001) and the ElMl group (63.6%, 9.1%, and 4.5%,respectively, P < 0.001) (Fig. 6a, b). The 1-, 3-, and 5-year DFS rates of the ElMl group were remarkably lower than those of the ElMh group (*P* = 0.003) and the EhMl group (41.7%, 33.3%, and 33.3%, respectively, *P* = 0.010). The 1-, 3-, and 5-year OS rates of the ElMl group were markedly lower than those of the ElMh group (81.8%, 54.5%, 40.9%, respectively, *P* = 0.002). Moreover, there was a significant difference in OS between the EhMh and ElMh group (P = 0.003) (Fig. 6a, b). Overall, the results indicated that the combination of elevation of both ELF and c-Myc in HCC tissues seems to be predictive of the best prognosis.

Finally, we observed the predictive effect of c-Myc expression combined with TGF-β1 and ELF for outcomes in patients with HCC through analysis of the following eight groups: TGF-β1 high/ELF high/c-Myc high expression (ThEhMh), TGF-β1/ELF low/c-Myc high expression (ThElMh), TGF-β1 high/ELF high/c-Myc low expression (ThEhMl), TGF-β1 high/ELF low/c-Myc low expression (ThElMl), TGF-β1 low/ELF high/c-Myc high expression (TlEhMh), TGF-β1 low/ELF high/c-Myc low expression (TlEhMl), TGF-β1 low/ELF low/c-Myc high expression(TlElMh), TGF-β1 low/ELF low/c-Myc low expression (TlElMl).

The 1-, 3-, and 5-year OS rates of the TlEhMh group (100%, 94.7%, 89.5%, respectively) were the best, and significantly higher than those of the ThEhMh (100.0%, 66.7%, 55.6%, respectively, *P* = 0.026), ThElMh (78.6%, 35.7%, 21.4%, respectively, *P* < 0.001), ThEhMl (57.1%, 14.3%, 14.3%, respectively, *P* < 0.001), TlElMl (100%, 0%, 0%, respectively, P < 0.001), and ThElMl groups (60.0%, 10.0%, 5.0%, P < 0.001). Similarly, the 1-, 3-, and 5-year OS rates of the TlElMh group (87.5%, 87.5%, 75.0%, respectively) were also remarkably higher than those of the ThElMh (P = 0.020), ThEhMl (P < 0.001), TlElMl (*P* = 0.029), and ThElMl groups (*P* < 0.001). The 1-, 3-, and 5-year OS rates of the TlEhMl group (80.0%, 80.0%, 60.0% respectively) were also higher than those of the ThElMl group (*P* = 0.011). The 1-, 3-, and 5-year OS rates of the ThEhMh group were also significantly higher than those of the ThEhMl (*P* = 0.038), and ThElMl group (*P* = 0.001). However, there was no significant difference of OS between the other two groups (*P* > 0.05).

**Fig. 7:**
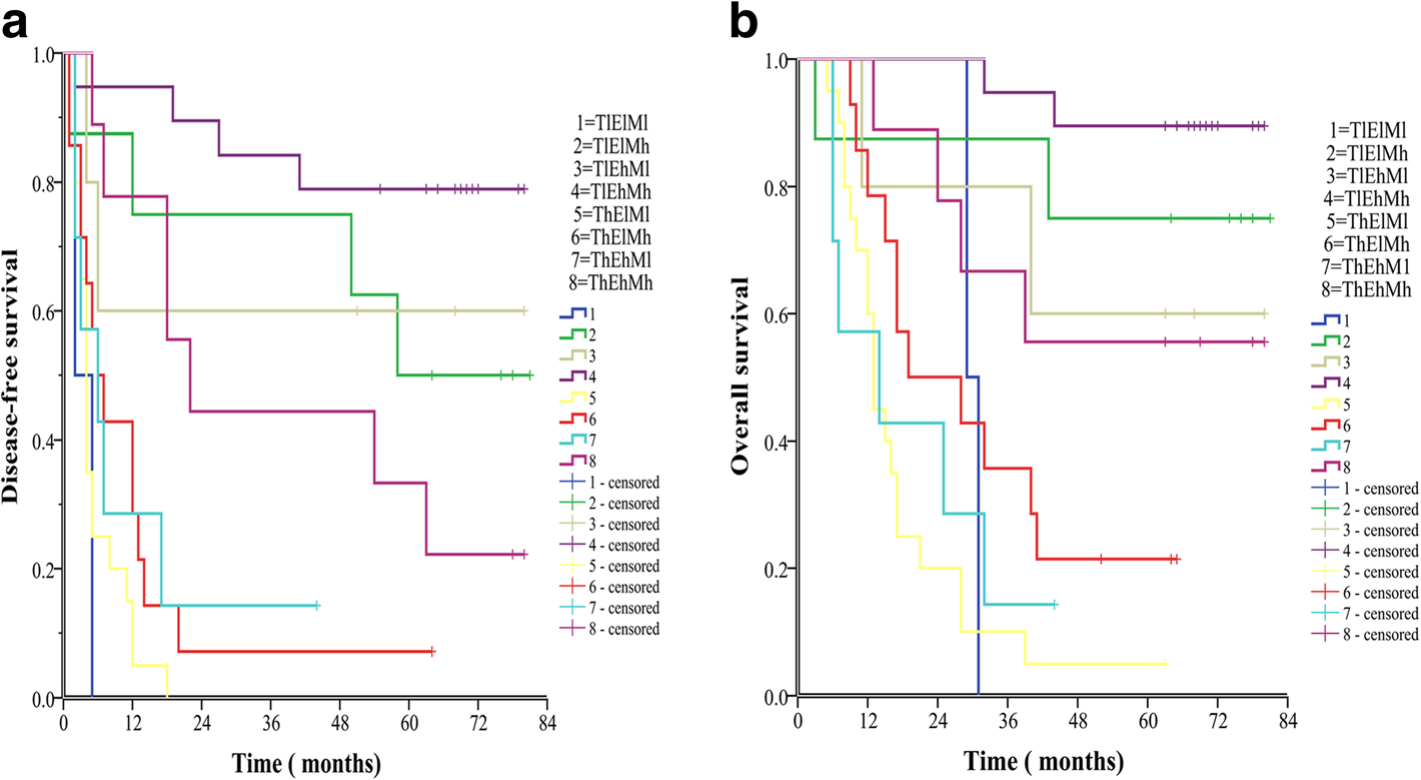
The combination of c-Myc, TGF-β1 and ELF was found to enhance prognostic accuracy for HCC. Disease-free survival curves (**a**, *P* < 0.001, log-rank test) and overall survival curves (**b**, *P* < 0.001, log-rank test)

The 1-, 3-, and 5-year DFS rates of the TlEhMh group (94.7%, 84.2%, 78.9%, respectively) were significantly higher than those of the ThEhMh (77.8%, 44.4%, 33.3%, *P* = 0.003), ThEhMl (28.6%, 14.3%, 14.3%, respectively, P < 0.001), ThElMh (28.6%, 7.1%,7.1%, respectively, P < 0.001), ThElMl (5.0%, 0.0%, 0.0%, respectively, P < 0.001), and TlElMl groups (0%, 0%, 0%, respectively, P < 0.001). The 1-, 3-, and 5-year DFS rates of the TlElMh group (75.0%, 75.0%, 50.0%, respectively) were also significantly higher than those of the ThEhMl (*P* = 0.024), ThElMh (*P* = 0.009), ThElMl (P < 0.001), and TlElMl (P = 0.029) groups. Likewise, the 1-, 3-, and 5-year DFS rates of the TlEhMl group (60.0%, 60.0%, 60.0%, respectively) were also significantly higher than those of the ThElMl (*P* = 0.007) and TlElMl group (*P* = 0.057). The 1-, 3-, and 5-year DFS rates of the ThEhMh group were also significantly higher than those of the ThEhMl (*P* = 0.037), ThElMh (*P* = 0.021), ThElMl (P < 0.001), and TlElMl (*P* = 0.004) groups. However, there was no obvious difference of DFS between the other two groups (P > 0.05) (Fig. 7).

## Discussion

Undoubtedly, c-Myc plays an important role in the occurrence and development of HCC. For example, overexpression of the c-Myc gene has been frequently observed in human HCC tissues. And c-Myc has induced the occurrence of HCC in animal models. Accordingly, c-Myc inhibition results in a loss of the neoplastic properties [18–21]. However, c-Myc also exhibits onco-suppressive activity, such as apoptosis and cell senescence [12, 17, 22, 23]. Kaposi-Novak et al. [27], Akita et al. [28] and Murphy et al. [29] have shown that when c-Myc was at a low level, oncogenic properties were increased and proliferation was induced, once c-Myc exceeded a threshold level, the upregulation of pro-apoptotic factors p53, p21, and p27 and cell death from apoptosis were detected.

There are few studies about the level of c-Myc protein in human HCC and adjacent tissues, or about the prognostic value of c-Myc protein in HCC. Cui et al. [35] found that there was higher c-Myc expression in HCC tissues with tumor recurrence within 1 year after tumor resection (15 cases), compared with that in HCC tissues without tumor recurrence (15 cases), and made a conclusion that high levels of c-Myc protein was related to a high recurrence rate. However, Yuen et al. [36] drew a completely different conclusion by analyzing the data of 150 patients with HCC. They found that the expression of c-Myc protein was negatively correlated with the grade of tumor differentiation and the levels of mutated p53.

Our results showed that there was low expression of c-Myc protein and mRNA in normal liver tissue, while there was high expression in HCC and adjacent tissues. We also observed a phenomenon reported by Yuen et al. [36] that there was the higher expression of c-Myc protein and mRNA in adjacent tissues as compared to those of HCC tissues. Furthermore, we found that c-Myc protein in HCC tissues was negatively correlated with PVTT, tumor number, tumor size, and recurrence, whereas there were no correlation between clinicopathological characteristics and c-Myc expression in adjacent tissues except complete tumor encapsulation.

We further analyzed the clinical significance of c-Myc protein in the outcomes of patients with HCC, and found that the expression of c-Myc protein was a significant and independent prognostic factor for DFS and OS, and the predictive accuracy was better than that of TNM stage, another independent prognostic factor for HCC. The results showed that low expression of c-Myc protein predicts recurrence and the poor outcomes in patients with HCC. The 1-, 3-, and 5-year DFS and OS rates (70.0%, 54.9%, 45.9% and 92.0%, 72.0%, 62.0%, respectively) in patients with HCC with high c-Myc expression were remarkably higher than those with low c-Myc expression (17.6%, 11.8%, 11.8% and 64.7%, 20.6%, 14.7%).

It is well known that the recurrence of HCC after resection is intimately associated with a decreased median survival. Moreover, the recurrence within the first postoperative year predicted a worse prognosis [37]. We observed that low c-Myc expression in HCC tissues was also intimately associated with short-term recurrence and a lower OS rate, compared to those with the long-term recurrence and no recurrence. The proportion of low c-Myc expression in patients with HCC with recurrence at ≤1 year was 62.8%, which was remarkably higher than that in patients with HCC with a recurrence after 1 year and no recurrence (14.3% and 14.8%, respectively).

Overexpression of TGF-β1 is usually found in HCC tissues, and it correlates with carcinogenesis and progression of HCC. Disruption of TGF-β signaling is recognized as the key mechanism through which the function of TGF-β is changed from tumor suppression to tumor promotion. Santoni-Rugiu et al. [33] indicated that c-Myc induced HCC carcinogenesis was strengthened by TGF-β1 overexpression. Our previous studies [30, 32] showed high-expression of TGF-β1 in rat HCC tissues, and abnormality of the TGF-β1 signaling pathway might promote the carcinogenesis of HCC, also indicated that a high level of TGF-β1 foreboded a poor prognosis of in patients with HCC after curative resection. The loss or down-regulation of ELF (a Smad3/4 adaptor protein) might be one of reasons for disruption of TGF-β1 signaling, which similarly predicted a poor OS and DFS rate for patients with HCC. Interestingly, their combination had a better prognostic value, compared with either one alone.

In this study, we analyzed the correlation of c-Myc with TGF-β1 and ELF, and found that c-Myc was negatively associated with TGF-β1 and positively with ELF. Intriguingly, the combination of c-Myc with ELF or/and TGF-β1 increased predictive accuracy for outcomes in patients with HCC who underwent resection, and in particular the combination of all three.

Our results showed that patients with HCC with the best prognosis had low TGF-β1 and high c-Myc expression (3-year OS and DFS rates of 92.6% and 81.5%, respectively), while the worst prognosis was seen in patients who have high TGF-β1 and low c-Myc expression (3-year OS and DFS rates of 11.5% and 3.8%, respectively). The second best was in patients of low TGF-β1 and low c-Myc expression (3-year OS and DFS rates of 42.9% and 42.9%, respectively), and the third was in patients of high TGF-β1 and high c-Myc expression (3-year OS and DFS rates of 47.8% and 21.7%, respectively). So we can conclude that high TGF-β1 or low c-Myc expression both predict poor prognosis in patients with HCC, and this combination is associated with the poorest outcomes. In addition, the prognostic value of TGF-β1 may be better than that of c-Myc for patients with HCC with hepatectomy.

As for ELF and c-Myc, we found that patients with HCC with high ELF expression and high c-Myc expression had the best prognosis (3-year OS and DFS rates of 85.7% and 71.4%, respectively), while the worst prognosis was seen in those with low ELF and low c-Myc expression (3-year OS and DFS rates of 9.1% and 0%, respectively). The second best was in patients of low ELF and high c-Myc expression (3-year OS and DFS rates of 54.5% and 31.8% respectively), and the third was in patients with high ELF and low c-Myc expression (3-year OS and DFS rates of 41.7% and 33.3%, respectively). So we can conclude that low ELF expression or low c-Myc expression both predict a poor prognosis, and the worst outcomes are seen with low levels of both. In addition, the prognostic value of c-Myc may be better than that of ELF for patients with HCC with curative resection.

When all three markers were examined together, the worst outcomes were seen in patients with any combination of two or more of high TGF-β1, low ELF, and low c-Myc expression. The sequence of prognostic value of the three proteins for patients with HCC was TGF-β1 > c-Myc > ELF. The prognosis of patients of low TGF-β1, high ELF, and high c-Myc expression was the best (3-year OS and DFS rates of 94.7% and 84.2%, respectively). The second best was seen in patients of low TGF-β1, low ELF, and high c-Myc expression (3-year OS and DFS rates of 87.5% and 75.0%, respectively), the third was in patients of low TGF-β1, high ELF, and low c-Myc expression (3-year OS and DFS rates of 80.0% and 60.0%, respectively), and the fourth was in patients of high TGF-β1, high ELF, and high c-Myc expression (3-year OS and DFS rates of 66.7% and 44.4%, respectively).

Based on our results, we speculate that c-Myc protein may play an important role in the process of HCC carcinogenesis, rather than sustaining the growth of the tumor cells. It may be one of the contributory mechanisms of HCC progression, i.e., the apoptosis of HCC cells is jeopardized by the diminished expression of c-Myc and accompanying uncoordinated control of cellular growth. Low expression of c-Myc predicts short-term recurrence and a poor outcome in patients with HCC, though there is c-Myc expression in most HCC tissues, whereas there is no c-Myc expression in normal liver tissues. c-Myc combined with TGF-β1 or/and ELF can more accurately predict the prognosis in patients with HCC, given the disruption of the TGF-β1 signaling pathway is another important mechanism of HCC development. The combination of TGF-β1, ELF, and c-Myc expression is a valuable predictive method for outcomes in patients with HCC, which can usefully guide the follow-up and the further treatment for patients with HCC after curative resection. For instance, close follow-up should be performed to detect early tumor recurrence for the patients with any two or all of high TGF-β1, low ELF, and low c-Myc expression in tumor tissues. Accordingly, the personalized comprehensive therapies should be provided for this kind of patients, such as TACE, chemotherapy, sorafenib and percutaneous ablation.

Certainly, this study still has several limitations and unanswered questions. First, our present study is a retrospective, single-institution study with a relatively small number of patients. A prospective and well-designed study with larger number of patients with HCC with radical surgery is needed. Second, why does the level of c-Myc expression in HCC tissues were lower than those in adjacent tissues? Why was the level of c-Myc in HCC tissues not correlated with TNM stage? Whether c-Myc plays the important roles on carcinogenesis and early stage of HCC? Thus, it is worthwhile to perform further studies to verify our findings and test their clinical application for patients with HCC.

## Conclusion

Our study demonstrates that low expression of c-Myc protein predicts poor outcomes in patients with HCC with hepatectomy. The combination of TGF-β1, ELF and c-Myc can be used to accurately predict outcomes in patients with HCC. From a diagnostic point of view, our results prompt that the detection of c-Myc alone or combining with ELF/ TGF-β1 in tumor tissues could be a new prognostic marker in patients with HCC. We can depend on them to provide the personalized comprehensive therapies for patients with HCC, especially patients with any two or all of low c-Myc, high TGF-β1, and low ELF expression in tumor tissues. Further studies should be conducted to confirm our hypothesis that c-Myc proteins play an important role on hepato-carcinogenesis and early stage of HCC, and to elucidate the critical mechanisms of c-Myc in HCC.

## Abbreviation

AFP: Alpha-fetoprotein
CI: Confidence interval
CT: Computed tomography
DFS: Disease Free survival
ELF: Embryonic Liver Fodrin
HBsAg: Hepatitis B surface antigen
HCC: Hepatocellular cancer
IRS: Immunoreactivity score
MRI: Magnetic resonance imaging
OS: Overall survival
PLT: Platelet
PVTT: portal vein tumor thrombi. HR: Hazard ratio
qRT-PCR: Real -time Quantitative Polymerase Chain Reaction
TACE: Transcatheter arterial chemoembolization
TGF-β: Transforming growth factor β
TNM: Tumor-node-metastasis
